# Blindness and the Midwife

**Published:** 1914-04

**Authors:** 


### Blindness and the Midwife.

In thirteen States of the Union there are absolutely no restrictions upon practice by midwives; in fourteen more it has not been possible to ascertain any statutory requirement for their training, licensure, or control; in two only does legislation approach what is right and proper. The Committee for the Prevention of Blindness sent their secretary, Miss Carolyn C. Van Blarcom, to Europe to ascertain what had been accomplished there, and we have before us part of her experiences recorded in 141 pages of "The Midwife in England," with an introduction by Dr. J. Clifton Edgar, published at 130 East Twenty-second Street, New York. The present condition in America seems to the author to be summarized by a beldame, eighty years of age, who declared, "I am too old to clean; too weak to wash; too blind to sew; thank God, I can still put my neighbor to bed!" Such a woman will assure her patients that sore eyes are natural in a baby, will prescribe milk, lemon juice, lard, raw potatoes, scraped beef, saliva, etc., and when the baby goes blind, piously declare that it is the will of God. Owing to the character of recent immigration, over 40 per cent of women in America are now attended in childbirth by midwives, yet there is but one training school in the whole country, that at Bellevue Hospital.

The present requirements in England are briefly as follows: A candidate must, under satisfactory supervision, have attended and watched the progress of not fewer than twenty labors, she must have nursed twenty lying-in women and their infants during the ten days following labor, and she must have attended a course of instruction, extending over not less than three months and consisting of not fewer than fifteen lectures, in elementary anatomy, pregnancy and its complications, including abortion, signs of abnormal labor, hemorrhage, preparation of antiseptics, management of the patient, including use of thermometer and catheter and taking of the pulse, management of the infant for ten days, including feeding and signs of disease, duties of a midwife as described in the regulations—to observe which demands intelligence and care—obstetric emergencies, puerperal fever, effects of venereal disease on the newborn, disinfection of person, clothing, and apparatus, principles of hygiene as regards home, food supply, and person, and the care of children apparently lifeless. Many graduate nurses add the midwife's training to their own in order to qualify for certain posts. Three of the forty English schools give free tuition, and in a few instances the cost runs up to $145. The examination is strict, both written and oral, defects in training being frequently discovered by one test when the other has been passed satisfactorily.

Owing to our Constitution this matter must be taken up by the States separately, and it is complicated by such problems as the immense number of foreigners with their own customs and superstitions, the division into white and black, the very size of our country indeed. It demands immediate and sustained attention, for fitness or unfitness in a midwife may mean life or death, sight or blindness, health or invalidism, physical well being or life-long misery for untold numbers of mothers and children. There could not be better work for committees of women than to urge the necessary steps on the various Legislatures, and we commend this booklet most earnestly to the attention of such committees, for it contains important details to which our limited space has prevented more than the barest reference. If object lessons are needed, a few visits to asylums for the blind should serve to fire the average intelligent woman with an unquenchable enthusiasm for so necessary and so appealing a crusade.—*New York Medical Journal Editorial.*

